# Assessing the impact of sampling bias on node centralities in synthetic and biological networks

**DOI:** 10.1038/s41540-025-00526-w

**Published:** 2025-05-15

**Authors:** Ali Salehzadeh-Yazdi, Marc-Thorsten Hütt

**Affiliations:** School of Science, Constructor University, Bremen, Germany

**Keywords:** Complexity, Computer modelling, Systems analysis

## Abstract

Centrality measures are crucial for network analysis, offering insights into node importance within complex networks. However, their accuracy is often affected by observational errors and incomplete data. This study investigates how sampling biases systematically impact centrality measures. We simulate six types of biased down-sampling, transitioning networks from dense to sparse states, using the initial network as the ‘ground truth.’ Changes in centrality values reveal the robustness of these measures under various sampling scenarios across synthetic and biological networks. Our results show that in synthetic networks, some sampling methods consistently exhibit higher robustness, particularly in scale-free networks. For biological networks, protein interaction networks are the most robust, followed by metabolite, gene regulatory, and reaction networks. Local centrality measures generally show greater robustness, while global measures are more heterogeneous and less reliable. This study highlights the limitations of centrality measures under sampling biases and informs the development of more robust methodologies.

## Introduction

The connectivity patterns between nodes in complex networks can reveal crucial information about the significance of each node with centrality indices being a prominent quantifier of such connectivity patterns. These indices provide a set of mathematical metrics to assess the importance of nodes within complex networks, facilitating the identification of influential nodes across a wide range of applications, including social media for identifying strong information spreaders ‘influencers’^1^, marketing to target campaigns^2^, search engines for ranking websites^3^, infectious disease analysis to track disease spreading^4^, neuroscience research to identify key regions for cognition and behavior^5^, and biological systems for predicting essential genes, drug targets and biomarkers^6^.

While classical measures such as degree, betweenness, closeness, and eigenvector centrality remain prevalent, extensive research has led to the development of over 100 centrality indices, each offering unique perspectives on the significance and influence of nodes within complex networks^7^. This evolution of centrality measures has not only expanded the field beyond its social network roots^8^ but has also enabled a deeper exploration of increasingly complex biological networks. As a result, researchers can now investigate the intricate dynamics of node importance and influence within these systems^9,10^.

Among the pioneering applications in biological networks was the centrality–lethality rule, introduced by Jeong et al., which established a correlation between connectivity and indispensability in yeast protein interaction networks (PINs)^11^. Following this seminal work, numerous studies have applied centrality indices to examine the structure and properties of diverse biological networks, from *E. coli* metabolic networks^12^ to gene regulatory networks^13^. These studies have revealed small-world features and power-law distributions, shedding light on the fundamental characteristics of biological systems and their potential for predicting the behavior of individual nodes within networks.

Any (network functional) insights such centrality indices can provide are ultimately limited by the completeness and accuracy of the network structure. In real-world networks, such as PINs, which are often incomplete and subject to errors^14–16^, accurately determining the centrality of nodes is challenging^17^. Sampling bias (also often termed observation bias or observational error) summarizes the range of distortions due to a non-random distribution of measurements, when data are accumulated over time^18^. In network representations this leads to incomplete networks with distorted structural features. This can be due to a variety of factors: experimental limitations, artifacts, or uncertainty in the analysis. For instance, in the case of PINs, experimental limitations (e.g., limited detectability and bait selection bias) contribute to this distortion. Weak interactions and researchers’ focus on specific proteins can lead to underrepresentation of certain interactions and overrepresentation of others^19^. Remarkably, only a small fraction of proteins (~5000) attracts the majority of research focus, leaving countless others unexamined^20^.

The effects of sampling bias on centrality measures can be significant and multifaceted. Sampling bias can introduce inaccuracies that significantly impact the calculation of centrality indices by distorting the overall connectivity patterns^18^. This distortion can lead to changes in centrality rankings, as the removal of critical connections or influential nodes can artificially affect the importance of other nodes. Different sampling methods introduce varying levels of bias, which can affect the network structure and centrality indices in distinct ways.

Despite the growing recognition of observational error as a prevalent concern in network analysis, a pervasive tendency to disregard its implications has impeded the development of robust methodologies to address this issue and potentially compromised the credibility of network-based predictions^21^. Several studies have attempted to address the challenges posed by observational error in network analysis, particularly in social and random networks. In 2003, Costenbader and Valente aimed to understand the stability of centrality measures in social networks under different sampling conditions. The study evaluated the stability of 11 different centrality measures and found that some were more stable than others, depending on the network’s properties^22^.

In 2005, Frantz and Carely demonstrated that centrality measures for core-periphery networks were highly sensitive to even small error levels, in contrast to uniform and cellular topologies. As error increased, all three topologies exhibited a decline in robustness, eventually converging to a similar level^23^.

In 2006, Borgatti et al. examined the impact of random errors on the accuracy of centrality measures in networks of varying sizes and densities. The results showed that centrality measures become less accurate as the amount of error increases, and that different centrality measures respond similarly to errors. Dense networks were more robust to errors, except in the case of edge deletion, where sparse networks were more accurately measured^24^.

Martin and Niemeyer^25^ addressed the issue of measurement errors in network studies by developing a method to estimate their impact on centrality measures. Experiments on random and real-world networks validated the effectiveness of the method’s ability to approximate robustness. The paper also highlighted the vulnerability of eigenvector centrality compared to PageRank in specific networks. They also found that networks with a higher average degree are often more robust^26^.

While several studies have addressed the challenge of observational error in social and random networks, biological networks have received less attention despite their crucial role in biomedical research. Few studies have investigated observational error in biological networks, including protein-protein interactions, gene regulatory networks, and metabolic pathways^27–29^. This gap in research is significant given the unique structural properties and functional implications of biological networks.

To address this research gap, we propose a novel approach: simulating various forms of sampling bias through biased down-sampling of network structures. Reading these results in the opposite direction then informs us, how centrality measures change under different sampling biases, and we can assess the robustness of different centrality measures under various error conditions. This approach provides unique insights into the behavior of these measures in incompletely observed networks, particularly in biological contexts.

**Fig. 1:**
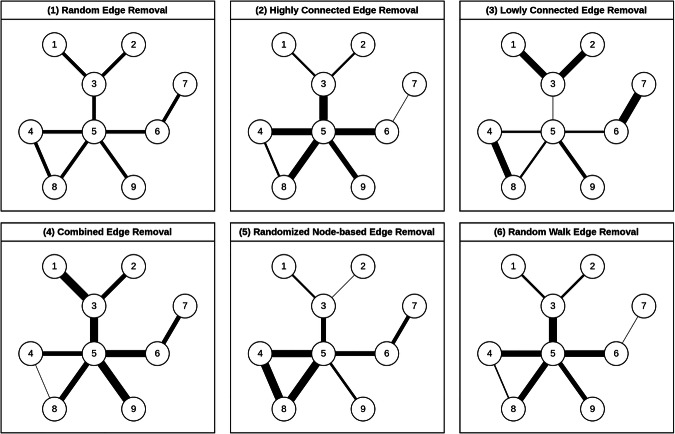
Illustration of six edge removal methods used to simulate observational errors in network structures. Edge thickness indicates the likelihood of removal. 1. Random Edge Removal (RER): Unbiased random deletion of edges. 2. Highly Connected Edge Removal (HCER): Targets edges linked to high-degree nodes, affecting network hubs. 3. Lowly Connected Edge Removal (LCER): Removes edges from low-degree nodes, minimally impacting core structures. 4. Combined Edge Removal (CER): Focuses on edges from nodes with extreme degree values, balancing central and peripheral effects. 5. Randomized Node-based Edge Removal (RNBER): Uses random node values to determine edge removal, introducing node-level variability. 6. Random Walk Edge Removal (RWER): Deletes edges based on their selection probability during random walks, simulating biased information collection.

We developed and simulated six stochastic edge removal methods to emulate the impact of potential observational errors: random edge removal (RER), highly connected edge removal (HCER), lowly connected edge removal, combined edge removal, RNBER, and random walk edge removal (RWER). These methods were chosen to represent a range of potential biases that might occur in real-world network observations. We then evaluated the stability of various centrality measures under these conditions across both synthetic and biological networks. Figure 1 provides a visual representation of these six edge removal methods, with thicker edges indicating a higher likelihood of removal. These methods introduce different disruptions to the network structure, affecting centrality rankings and revealing the robustness of these measures to various types of errors. By understanding the rationale behind each method, we can gain insights into the limitations and strengths of centrality metrics in network analysis.

Our findings reveal that the robustness of centrality measures varies depending on the type of network and the nature of the errors. Notably, PINs show particularly high resilience to edge removal. This work provides valuable insight into the reliability of network analysis, with important practical implications for fields such as biology, network science, and data science, particularly when working with incomplete or biased network data.

## Results

We conducted a broad study to investigate the robustness of centrality measures under various edge removal scenarios across diverse network types. Our analysis included synthetic networks (Erdős-Rényi, scale-free, and Watts-Strogatz) at three edge density levels, as well as biological networks (BioGRID, STRING, metabolite, reaction, and gene regulatory networks of yeast). We assessed the resilience of local (degree centrality), intermediate (subgraph centrality), and global (betweenness, closeness, eigenvector, and PageRank) centrality measures in the presence of incomplete network data.

### Characteristics of the networks

We constructed synthetic networks with similar sizes but varying densities. Erdős-Rényi networks are characterized by random connections, while scale-free networks exhibit a power-law degree distribution, with a few nodes having extremely high degrees and many nodes with lower degrees. Watts-Strogatz networks are a hybrid of random and regular networks, having a short-average path length and high local clustering. The characteristics of all networks, including the number of nodes and edges, are summarized in Table 1.

**Table 1:** Characteristics of the synthetic and biological networks used in this study

Network Type	Nodes	Edges	Description
**Synthetic Networks**			
Erdős-Rényi (*p* = 0.1)	1000	50,160	Random connections
Erdős-Rényi (*p* = 0.2)	1000	99,540	Random connections
Erdős-Rényi (*p* = 0.5)	1000	249,598	Random connections
Scale-Free (*m* = 0.1)	1000	47,500	Power-law degree distribution
Scale-Free (*m* = 0.2)	1000	97,900	Power-law degree distribution
Scale-Free (*m* = 0.5)	1000	250,000	Power-law degree distribution
Watts-Strogatz (*β* = 0.1)	1000	50,000	Hybrid of random and regular networks
Watts-Strogatz (*β* = 0.2)	1000	100,000	Hybrid of random and regular networks
Watts-Strogatz (*β* = 0.5)	1000	250,000	Hybrid of random and regular networks
**Biological Networks**			
STRING database	6394	986,995	PIN (combined score ≥ 150)
BioGRID database	6600	572,076	PIN
GRN	6881	194,779	Gene regulatory network
Metabolite network	2753	4080	Network of metabolites
Reaction network	1507	8136	Network of biochemical reactions

The table summarizes the number of nodes and edges for each network, along with a brief description of their properties. Synthetic networks were generated using the NetworkX library in Python, while biological networks were obtained from publicly available databases.

For the synthetic networks:

1. Erdős-Rényi networks: These networks were generated using the nx.erdos_renyi_graph function from the NetworkX library in Python with 1000 nodes and edge probabilities of 0.1, 0.2, and 0.5, resulting in 50160, 99540, and 249,598 edges, respectively.
2. Scale-free networks: These networks were created using the nx.barabasi_albert_graph function from the NetworkX library in Python with 1000 nodes and edge attachment probabilities of 0.1, 0.2, and 0.5, yielding 47,500, 97,900, and 250,000 edges, respectively.
3. Watts-Strogatz networks: These networks were constructed using the nx.watts_strogatz_graph function from the NetworkX library in Python with 1000 nodes and rewiring probabilities of 0.1, 0.2, and 0.5, producing 50,000, 100,000, and 250,000 edges, respectively.

For biological networks, we used:

1. **STRING Database**: This protein-protein interaction network contains 6394 nodes and 986,995 edges, filtered to include only interactions with a combined score ≥150.
2. **BioGRID Database**: This protein-protein interaction network contains 6600 nodes and 572,076 edges.
3. **Gene Regulatory Network (GRN)**: This network contains 6881 nodes and 194,779 edges, representing gene regulatory interactions.
4. **Metabolite Network**: This network contains 2753 nodes and 4080 edges, representing connections between metabolites.
5. **Reaction Network**: This network contains 1507 nodes and 8136 edges, representing biochemical reactions.

For the sake of better comparison and consistency across network types, directed networks were treated as undirected in this study, a simplification commonly employed in network science to focus on topological properties^30–32^.

### Robustness of centrality measures in synthetic and biological networks

**Fig. 2:**
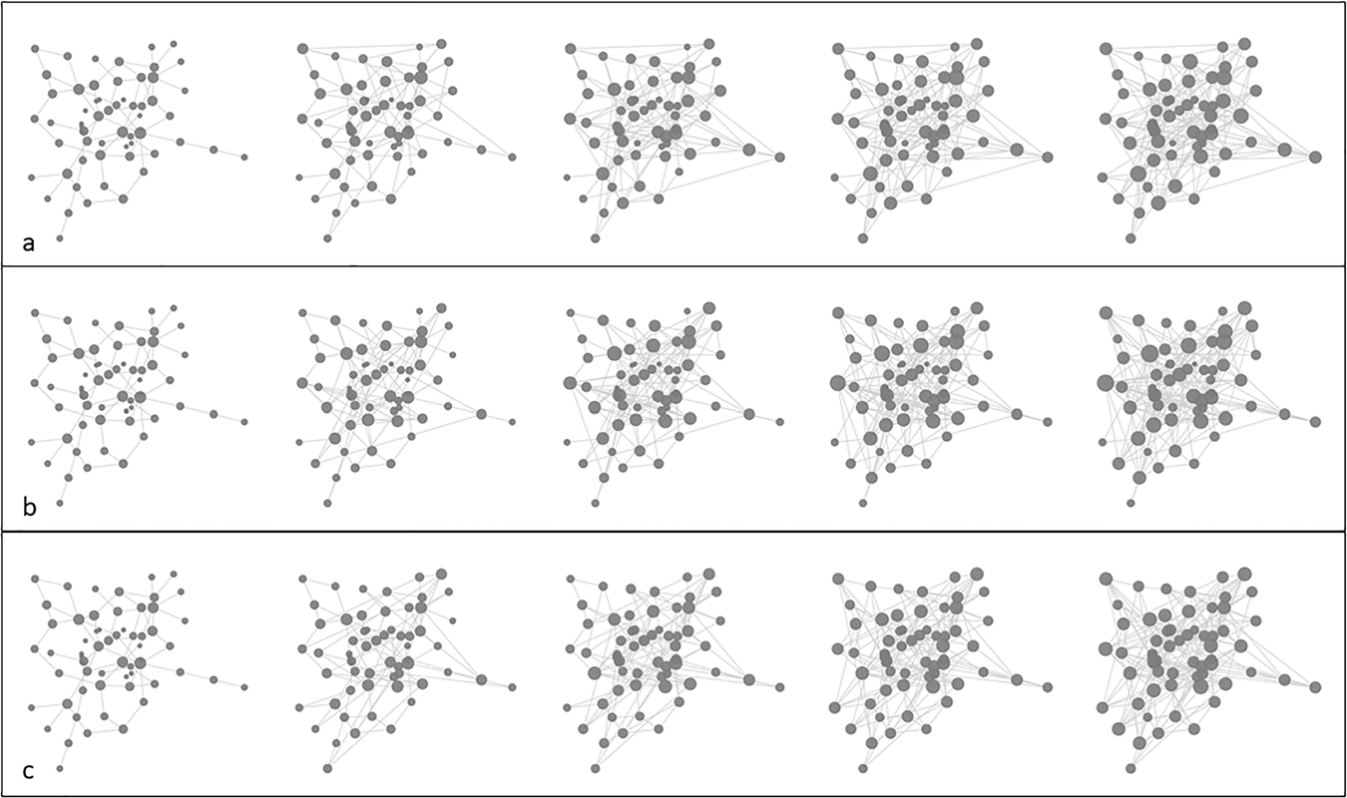
The series of networks depicts the gradual transformation of a sparse network into a highly connected graph due to the accumulation of observational errors. The node size represents the node’s connectivity, illustrating how observation errors can significantly alter network architecture. In (**a**) edges are added to the initial network randomly. In (**b**) highly connected nodes are more likely to receive new edges, while in (**c**), lowly connected nodes are more likely to receive additional edges.

**Fig. 3:**
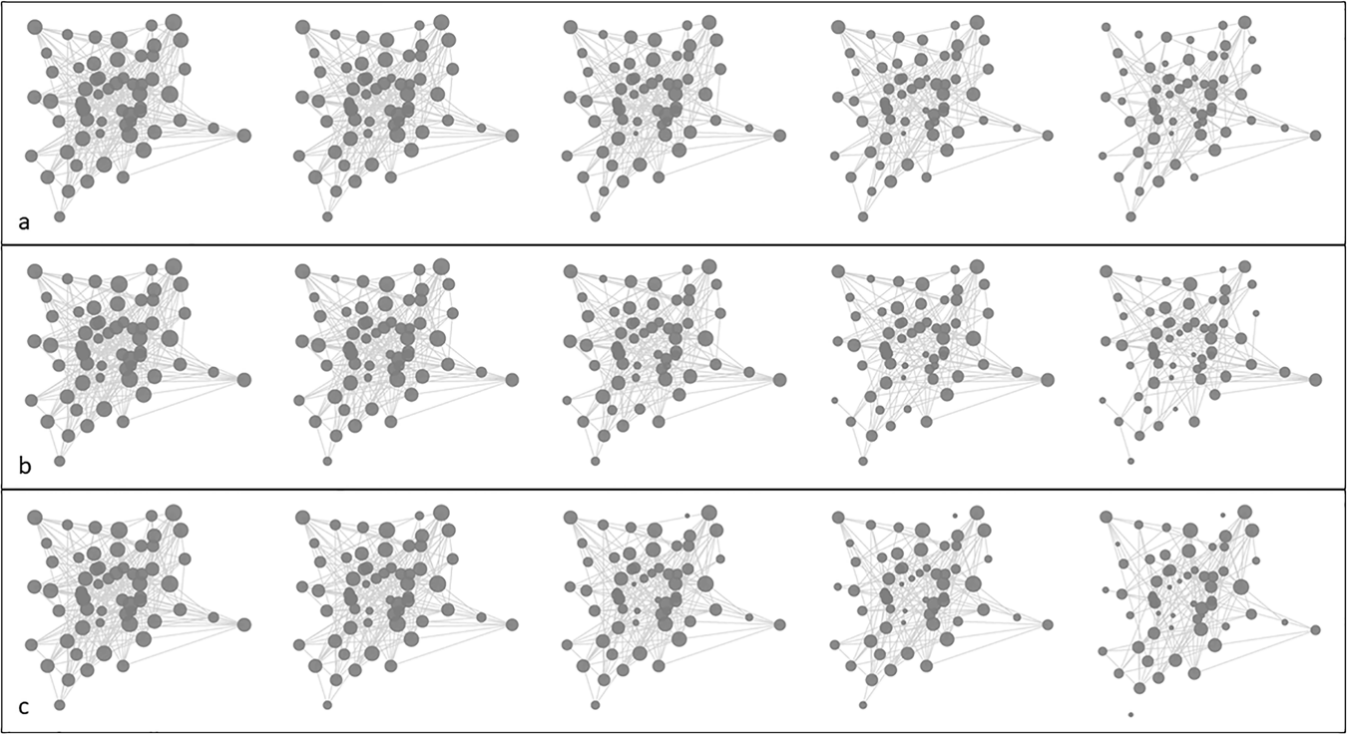
The sequence shows the transformation of a dense network into a sparse graph through three distinct edge removal mechanisms. The node size indicates connectivity strength, demonstrating how different types of edge removal mechanisms affect network structure. In (**a**) Random Edge Removal (RER) simulates general missing information by randomly removing edges. In (**b**) Highly Connected Edge Removal (HCER) preferentially removes edges from high-degree nodes, representing overestimation of important node connections. In (**c**) Lowly Connected Edge Removal (LCER) focuses on removing edges from low-degree nodes, simulating overlooked peripheral connections”.

Our study assumes that observational errors in networks manifest as a distorted sampling of the edge set of the ‘true’ network. Figure 2 illustrates the conceptual basis of our approach, showing how a sparse network gradually transforms into a highly connected graph due to the accumulation of more data, which can be biased by observational errors. To assess the robustness of centrality indices against these errors, we devised a reverse approach, simulating the removal of these erroneous links through six scenarios starting from current networks serving as (temporary) ‘ground truth’. This procedure thus leads to ever more incomplete networks. Figure 3 demonstrates how a dense network can become sparse through different edge removal processes. The node size represents connectivity, demonstrating how these errors can significantly alter network architecture.

**Fig. 4:**
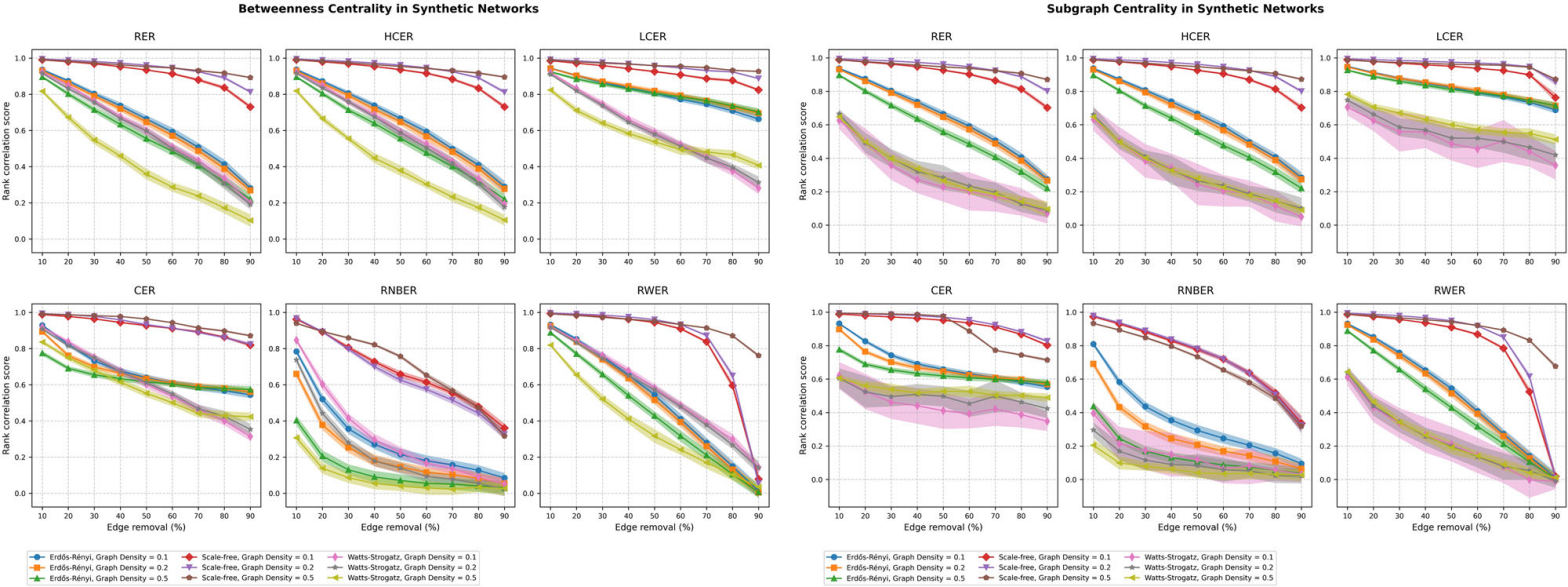
Impact of edge removal scenarios on betweenness and subgraph centrality in synthetic networks. (right panel) Changes in rank correlation of betweenness centrality under different edge removal scenarios. (left panel) Changes in rank correlation of subgraph centrality under the same edge removal scenarios. Each box represents a different edge removal scenario: Random Edge Removal (RER), Highly Connected Edge Removal (HCER), Lowly Connected Edge Removal (LCER), Combined Edge Removal (CER), Randomized Node-based Edge Removal (RNBER), and Random Walk Edge Removal (RWER). The x-axis represents the fraction of edges removed, while the y-axis shows the Spearman rank correlation coefficient between the centrality values in the original and perturbed networks. These examples illustrate how different centrality measures and removal scenarios can lead to varying patterns of network robustness.

To illustrate the effects of different edge removal scenarios on network centrality measures, we first present two examples in Fig. 4. The left panel shows the betweenness centrality, while the right panel displays the subgraph centrality, both for synthetic networks under all removal scenarios.

As evident from Fig. 4, different edge removal scenarios lead to varying impacts on centrality measures. For instance, the betweenness centrality (left panel) shows distinct patterns across scenarios, with some scenarios causing more rapid declines in rank correlation than others. Similarly, the subgraph centrality (right panel) exhibits unique behaviors under different removal conditions.

Building upon these illustrative examples, we present a comprehensive analysis in Supplementary Figure 1. In this figure, we organize the results into six panels, each corresponding to a specific centrality measure. To facilitate interpretation, we further divide each panel into two parts, showcasing the results for synthetic and biological networks separately. Within each panel, we illustrate the changes in rank correlation scores under various edge removal scenarios, highlighting the impact of each removal scenario, network structure, network density, and biological network type on these changes.

In terms of the impact of edge removal scenarios on changes in rank correlation scores, we observe consistent patterns across all centrality indices, with significant decreases in centrality robustness as the extent of edge removal increases. The exception to this trend can be seen in scale-free networks and PINs.

Concerning the impact of network structure and density on the robustness of centrality indices under various edge removal scenarios, our results indicate distinct patterns of centrality resilience among synthetic networks. As anticipated, scale-free networks generally exhibited the highest robustness, followed by Erdős-Rényi and Watts-Strogatz networks. In some instances, network density emerged as a contributing factor to increased resilience. However, the effects of edge removal approaches on resilience varied across different network types.

Unlike synthetic networks, biological networks did not follow a single, universal pattern of centrality robustness prioritization. Instead, each biological network type (BioGRID, STRING, gene regulatory networks, reaction networks, and metabolite networks) demonstrated unique resilience priorities. We found it necessary to examine each centrality measure individually, as the variation in the effects of edge removal approaches on resilience across different network types could occasionally alter the order of resilience.

**Fig. 5:**
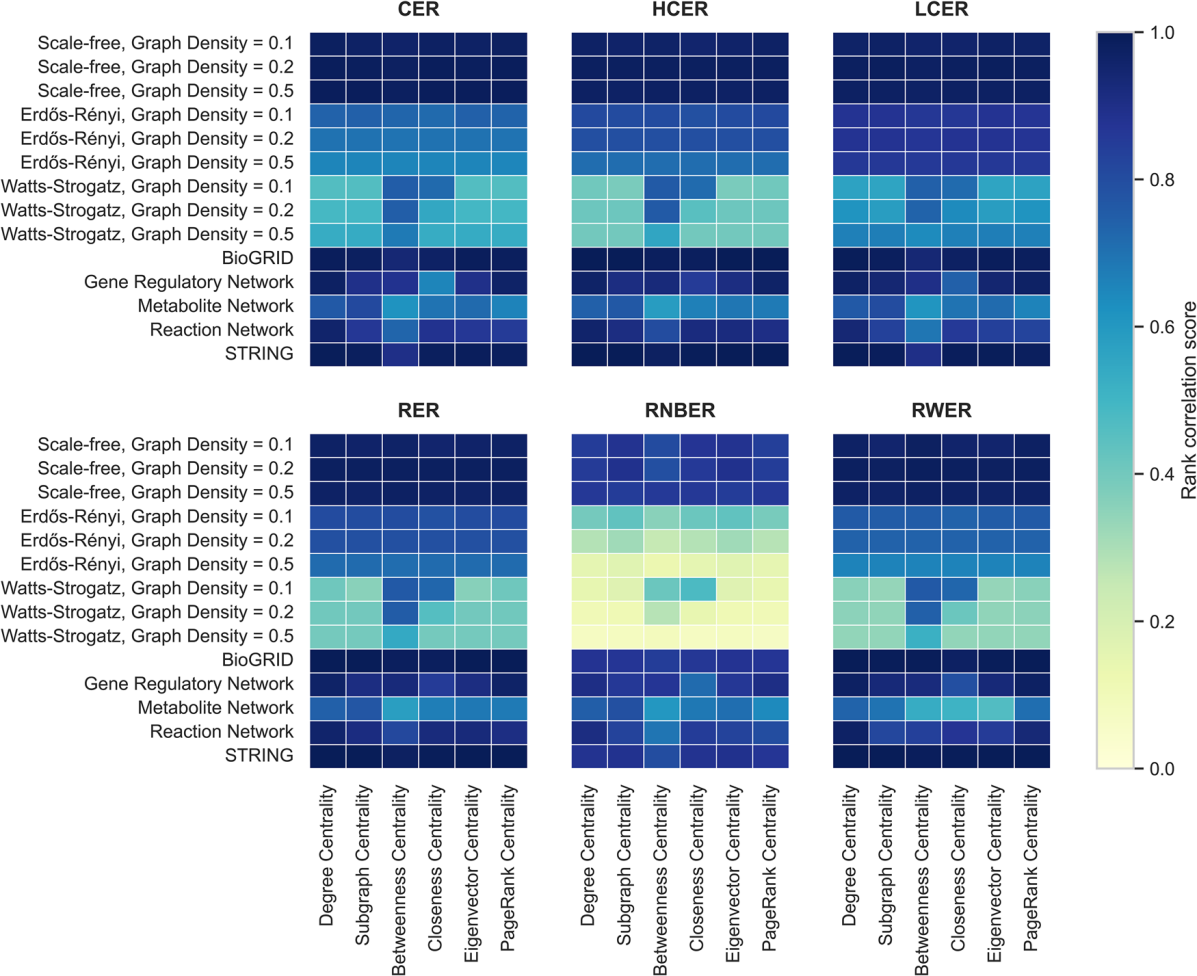
Heatmap visualization of rank correlation scores for 30% edge removal across various network types and centrality measures.

To facilitate the examination of each centrality measure and account for the variability in edge removal effects on resilience across network types, we generated a heatmap visualization. This heatmap (Fig. 5) presents rank correlation scores as different colors for 30% edge removal across all cases. By doing so, we aimed to simplify the interpretation of the complex results, enabling a more comprehensive understanding of the relationship between edge removal methods, network types, and centrality robustness.

The heatmap visualization effectively highlights the substantial differences among edge removal methods and their respective robustness. Mainly, LCER method demonstrates the highest robustness. This finding confirms that removing poorly connected edges has a relatively minimal impact on the overall network structure and centrality measure rankings. This is plausible, since low-degree nodes often have limited influence on the network’s connectivity patterns, allowing the network to maintain its stability despite their removal.

Conversely, the Randomized Node-based Edge Removal (RNBER) and RWER methods emerge as the most disruptive edge removal scenarios in our study. These methods, which correspond to systematic biases in experimental target selection, reveal critical insights into network vulnerability and the impact of biased data collection on network analysis.

The RNBER method, which assigns random values to nodes and removes edges based on these values, simulates scenarios where edge removal is influenced by node properties extrinsic to the network structure. This approach uncovers a significant vulnerability in networks: their susceptibility to disruption based on factors not inherently related to their topology. The substantial shifts in centrality measure rankings observed under RNBER highlight the potential for external biases to dramatically alter our understanding of a network’s structure and function.

Similarly, the RWER method, which removes edges based on their probability of being selected during a random walk, mimics the cascading effects of biased information collection. This scenario is particularly relevant in real-world research contexts, where investigating one node often leads to the exploration of its immediate neighbors, creating chains of experimental attention. Our findings show that this type of biased edge removal can significantly disrupt the network’s structure and stability. The marked changes in centrality rankings under RWER underscore how the path-dependent nature of many research processes can lead to skewed representations of network properties.

In analyzing the various centrality measures (Supplementary Fig. 2 and Supplementary Fig. 3), we did not observe significant distinctions among them. However, global centralities, like betweenness and eigenvector centrality, are less reliable, when biological networks are distorted by any forms of sampling bias. This suggests that in incomplete networks, the choice of centrality measure may not have a substantial impact on the overall results or predictions. Consequently, the robustness of these measures appears to be relatively consistent, indicating that missing data does not considerably influence their predictive capabilities in most cases. However, it is essential to consider that global centralities may be more sensitive to network incompleteness in some situations.

PINs consistently demonstrated the highest robustness among biological networks, with gene regulatory, reaction, and metabolite networks following in descending order. This finding underscores the need for caution when applying centrality measures to biological networks other than PINs. Due to their inherent complexity and unique structural characteristics, these networks may exhibit varying degrees of sensitivity to edge removal and missing data, which could potentially influence the accuracy and reliability of centrality-based predictions.

Conclusively, our results indicate that while local, intermediate, and global centrality measures exhibit similar performance in many contexts, there are differences when applied to biological networks. Specifically, global centrality measures tend to be less reliable when analyzing biological networks. Particularly, the way observational errors and missing data accumulate over time influences the overall robustness of the network, with global measures being more susceptible to these cumulative effects in biological systems. Local and intermediate centrality measures, which focus on more immediate network neighborhoods, appear to be more resilient to incomplete or biased data in biological networks. This resilience might be attributed to their ability to capture relevant information from smaller, more completely observed substructures within the larger, potentially incomplete network.

These findings suggest that researchers should carefully consider the unique properties of each network, when applying centrality measures. The interplay between network structure, edge removal scenarios, and centrality can significantly affect the reliability of predictions in the presence of incomplete data. For biological networks, it may be prudent to rely more heavily on local and intermediate centrality measures, or to develop hybrid approaches that balance the information provided by different types of centrality measures.

### Analysis of stability across centrality measures and stochastic edge removal methods: Application to yeast metabolic network

To evaluate the stability of centrality measures under different stochastic edge removal methods, we developed a systematic approach to identify stable nodes, which we define as metabolites whose rankings remain relatively consistent across varying levels of network perturbation.

For each centrality measure (degree, betweenness, closeness, PageRank, eigenvector, and subgraph centrality), we calculated the rank of each metabolite across networks generated under increasing levels of edge removal (10% to 90%). The standard deviation of these rankings was used as a measure of variability. Nodes with lower standard deviations were considered more stable, as their centrality rankings were less affected by the perturbations.

To quantitatively identify stable nodes, we determined a stability threshold, defined as the 25th percentile of the standard deviation values for each centrality measure. Metabolites with a standard deviation below this threshold were classified as stable nodes.

**Fig. 6:**
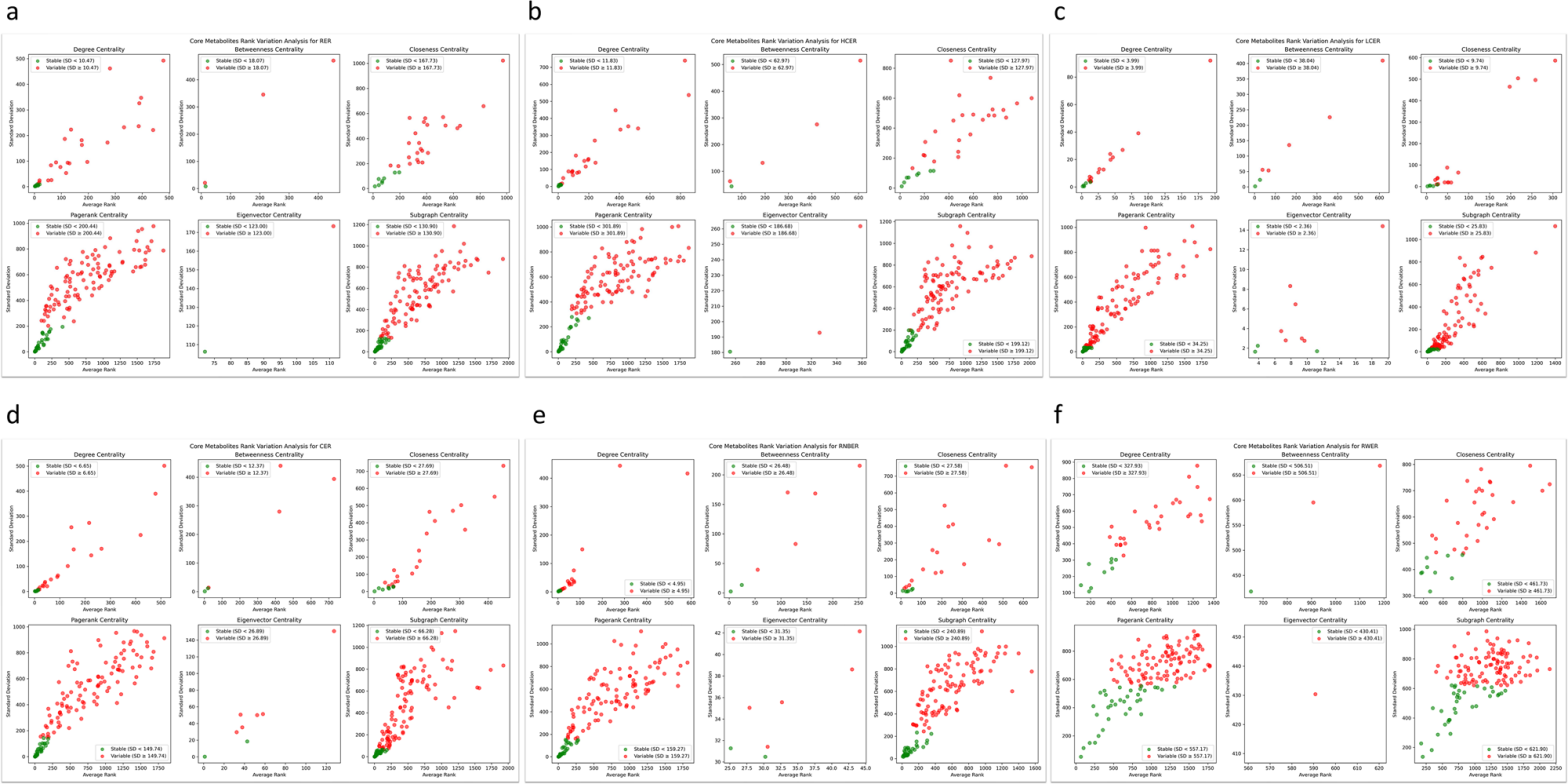
Stability analysis of centrality measures under six stochastic edge removal methods. **a** Random Edge Removal (RER), **b** Highly Connected Edge Removal (HCER), **c** Lowly Connected Edge Removal (LCER), **d** Combined Edge Removal (CER), **e** Randomized Node-based Edge Removal (RNBER), and **f** Random Walk Edge Removal (RWER). Each plot shows the variation in centrality rankings for metabolites across increasing levels of edge removal (10% to 90%). The x-axis represents the average rank, while the y-axis represents the rank variability (e.g., standard deviation) for each metabolite. Green color represents metabolites identified as stable nodes under the defined stability threshold for each centrality measure.

For each method, we analyzed the stability of centrality measures by identifying stable nodes under the defined stability threshold. The list of stable nodes for each centrality measure is provided in Supplementary Note 1. The percentage of stable nodes for each centrality measure presented in Table 2. Figure 6 highlights the trends in stability for each centrality measure under different perturbation scenarios.

**Table 2:** The percentage of stable nodes relative to all core metabolites for each centrality measure.table

edge removal methods	Stable nodes (%) for each centrality index					
	Degree	Betweenness	Closeness	Pagerank	Eigenvector	Subgraph
RER	25	25	25	25	50	25
HCER	25	20	25	25	33.33	25
LCER	25	28	25	25	30	25
CER	25	33.33	25	25	25	25
RNBER	26	28	26	25	28	25
RWER	25	33.33	24	25	0	25

The results demonstrate that the stability of centrality measures varies significantly depending on the edge removal method.

### Degree centrality

- Degree centrality was relatively stable across all methods, with stable nodes consistently identified as key metabolites (e.g., sM_glu_L_c, sM_nh4_c and sM_coa_x).
- The percentage of stable nodes ranged from ~24% to 26%, depending on the method.

### Betweenness centrality

- Betweenness centrality exhibited higher variability compared to degree centrality, with fewer stable nodes identified.
- For methods like HCER and RWER, stable nodes were limited to a small subset of metabolites, often those with critical roles in bridging network regions.
- The percentage of stable nodes ranged from 20 to 33%, highlighting the sensitivity of betweenness centrality to the type of perturbation.

### Closeness centrality

- Closeness centrality displayed moderate stability, with stable nodes often corresponding to metabolites central to the metabolic network (e.g., sM_glu_L_c, and sM_akg_c).
- The percentage of stable nodes was consistently 25% across most methods, indicating robustness to certain perturbations.

### PageRank centrality

- PageRank centrality was among the most stable measures, with stable nodes frequently including highly connected and biologically significant metabolites.
- The percentage of stable nodes was consistently around 25%, regardless of the edge removal method.

### Eigenvector centrality

- Eigenvector centrality showed mixed stability, with methods like RER and LCER identifying a higher percentage of stable nodes (~30–50%).
- This suggests that eigenvector centrality may be less sensitive to certain types of perturbations but more influenced by others.

### Subgraph centrality

- Subgraph centrality exhibited moderate stability, with stable nodes often overlapping with those identified by PageRank and closeness centrality.
- The percentage of stable nodes was consistently around 25%, similar to other centrality measures.

The application of our method to the yeast metabolic-centric network reveals biologically meaningful patterns. Stable nodes identified across centrality measures often correspond to key metabolites involved in fundamental metabolic processes, such as:

- sM_glu_L_ (L-glutamate): A central metabolite in amino acid metabolism.
- sM_nh4_c (Ammonium): Critical for nitrogen metabolism.
- sM_coa_x (Coenzyme A): Essential for energy and lipid metabolism.

We show that different methods lead to distinct stability profiles across centrality measures. The differences in stable nodes across methods further highlight how specific biases can influence the interpretation of network centrality. Our findings provide a framework for assessing the robustness of network analysis methods and highlight the need to account for potential biases when interpreting centrality-based results in biological networks.

## Discussion

The accurate assessment of node importance in complex networks is crucial for diverse applications, ranging from identification of influential individuals in social networks to critical genes in biological systems. Centrality measures, which quantify the significance of nodes based on their connections and connectivity patterns, serve as valuable tools for identifying key players in these networks. However, real-world networks are often incomplete and subject to errors in observation, making it challenging to accurately determine centrality rankings. Accordingly, it is pivotal to understand the resilience of centrality measures to network errors, as erroneous networks can lead to false conclusions and undermine the value of network analysis.

To address the inherent challenges of incomplete and error-prone network structures, we conducted a large-scale study to evaluate the robustness of centrality measures in the presence of observational error. Our primary objectives were to dissect and understand the intricate dynamics of network robustness through examination of various factors.

Firstly, we delved into the impact of different types of synthetic graphs, where we examined the influence of sampling scenarios on each type, including Erdős-Rényi, scale-free, and Watts-Strogatz networks, unraveling the varying degrees of robustness exhibited by each. Our exploration then extended to the impact of density within synthetic graphs, systematically assessing how variations in network density influence the overall robustness of our synthetic graphs. Furthermore, transitioning to real-world scenarios, we investigated the effects of these various scenarios on centrality robustness of PINs, encompassing BIOGRID, and STRING, metabolite, gene regulatory, and reaction networks of yeast.

Synthetic networks exhibited distinctive centrality resilience patterns. scale-free networks generally displayed superior robustness, followed by Erdős-Rényi and Watts-Strogatz networks, with network density occasionally influencing resilience. However, the impact of sampling approaches on resilience varied across network types.

In Erdős-Rényi networks, LCER and CER consistently demonstrated the highest robustness, retaining up to 70% of centrality ranking values even after 90% edge removal (Fig. S1). This highlights the efficacy of these methods in preserving centrality information in sparse networks. Similarly, in scale-free networks, LCER and CER also exhibited exceptional robustness, maintaining up to 80% and 90% of centrality ranking values after 90% edge removal. This exceptional performance is attributed to their ability to protect the network’s core, where most important connections reside. However, in Watts-Strogatz networks, the stability patterns of centrality measures were more heterogeneous. Betweenness and closeness centrality indices exhibited the highest robustness, while local and intermediate centralities displayed the lowest robustness. This suggests that betweenness and closeness are particularly well-suited for capturing the connectivity patterns in Watts-Strogatz networks.

In contrast, PIN networks generally showed higher robustness, with local centrality measures dominating in gene regulatory and metabolite networks. This indicates that local interactions and connectivity patterns play a more significant role in capturing the functional roles of nodes in these networks. Prioritizing centrality robustness in biological networks proved elusive, as each network type (BioGRID, STRING, gene regulatory, reaction, and metabolite networks) had unique priorities. The examination of individual centrality measures was necessary, and the impact of sampling approaches on resilience could alter the order of resilience. Results displayed assorted patterns of robustness across the diverse biological networks, emphasizing the need for tailored centrality approaches.

In summary, five key points emerged from this study:

1. **Network structure matters**: Scale-free networks and PINs show higher robustness to edge removal than the other networks in our study, highlighting the importance of considering network topology in centrality analysis.
2. **LCER vs. RNBER and RWER**: LCER is the most robust scenario, while RNBER and RWER are the most disruptive, suggesting that the nature of missing data significantly impacts network analysis.
3. **Centrality measure choice**: The choice of centrality measure has minimal impact on results in incomplete networks, except for betweenness and eigenvector in some cases.
4. **Biological network hierarchy**: Among biological networks, PINs demonstrate the highest robustness, followed by gene regulatory, reaction, and metabolite networks, indicating varying sensitivities to missing data.
5. **Caution in non-PIN biological networks**: Researchers should exercise caution when applying centrality measures to biological networks other than PINs, as they may be more sensitive to edge removal and missing data.

These findings collectively emphasize the importance of carefully considering the various factors that can impact the validity and reliability of network analysis and underscore the need for careful design and execution of network studies when evaluating the resilience of centrality measures and prioritizing robustness.

While our study primarily focuses on synthetic and yeast biological networks, the implications of our findings extend beyond these specific network types. The robustness of centrality measures under sampling bias is a fundamental concern in network science. Networks from diverse domains, such as social networks, power grids, and transportation networks, exhibit unique structural properties that may influence the behavior of centrality measures under observational errors. For instance, social networks often display strong community structures and high clustering coefficients, whereas power grids are characterized by their sparse and hierarchical structure. Investigating these networks under similar sampling scenarios could provide valuable insights into both universal patterns and domain-specific differences in centrality robustness. Future studies could expand on our framework by incorporating such networks to validate and generalize our findings. This expansion would not only strengthen the applicability of our results but also offer a deeper understanding of how sampling biases impact centrality measures in networks with distinct topologies and functional roles. By exploring these additional contexts, researchers can develop more robust methodologies tailored to the unique challenges posed by different network types.

Similarly, while the edge removal methods employed in this study offer valuable insights into the effects of sampling bias on centrality measures, they represent simplified models of real-world bias scenarios. In practice, sampling biases in networks often arise from complex and context-specific factors, such as experimental limitations, selective reporting, or systemic biases in data collection processes. Future research should aim to design edge removal strategies that more accurately reflect these real-world conditions by integrating empirical data and domain-specific bias features. For example, incorporating experimentally observed patterns of missing data could enhance the realism and applicability of the simulations. Additionally, refining existing methods by combining multiple bias features or developing hybrid approaches could further improve the practical significance of the results. These advancements would enable a more comprehensive understanding of how sampling biases influence network analysis and contribute to the development of more robust methodologies for real-world applications.

Finally, in this study, directed networks were simplified by treating them as undirected graphs to ensure consistency with the protein-protein interaction network (PIN), which is inherently undirected. While this simplification allows for a uniform comparison of centrality robustness across network types, it may overlook the degrees introduced by directional information, particularly in metrics such as in-degree and out-degree centrality. Future research should explicitly incorporate directionality to assess its impact on centrality measures and investigate whether the observed trends in centrality robustness under sampling bias hold for directed networks. Such an analysis would provide a more comprehensive understanding of how network topology influences the behavior of centrality measures in both directed and undirected contexts.

## Methods

### Experimental design and statistical analysis

For each edge removal scenario, we followed this procedure:

1. Calculate the specified centrality measure for the original network.
2. Remove 10% of the edges and recalculate the centrality.
3. Compute the Spearman correlation coefficient between the original and new centrality values. Note that the Spearman correlation of the centralities is the same as the Pearson correlation of the rank of these centrality values.
4. Repeat steps 2–3 for 20%, 30%, 40%, 50%, 60%, 70%, 80%, and 90% edge removal.
5. Replicate the entire process 10 times for each centrality measure and network.
6. Average the 10 Spearman correlation coefficients to obtain the final robustness measure.

### Datasets

We employed three types of synthetic networks (Erdős-Rényi^33^, scale-free^34^, and Watts-Strogatz^35^ models) and four biological networks of yeast, representing different levels of cellular organization: protein-protein interaction, gene regulatory, metabolite and reaction networks.

Synthetic networks were constructed using the NetworkX library^36^ in Python. Yeast PINs were extracted from BioGRID^37^ and STRING^38^ databases, while the gene regulatory network was obtained from published sources^39,40^. The genome-scale metabolic model of yeast was retrieved from the BiGG database^41^ (http://bigg.ucsd.edu/models/iMM904) and converted into metabolite and reaction networks using GEMTractor^42^.

### Diversity of centrality indices

We analyzed six centrality measures, categorized into three groups based on the topological scale evaluated in their computation^43^:

1. Local scale: Degree centrality.
2. Intermediate scale: Subgraph centrality.
3. Global scale: Betweenness, Closeness, Eigenvector, and PageRank centralities.

**Fig. 7:**
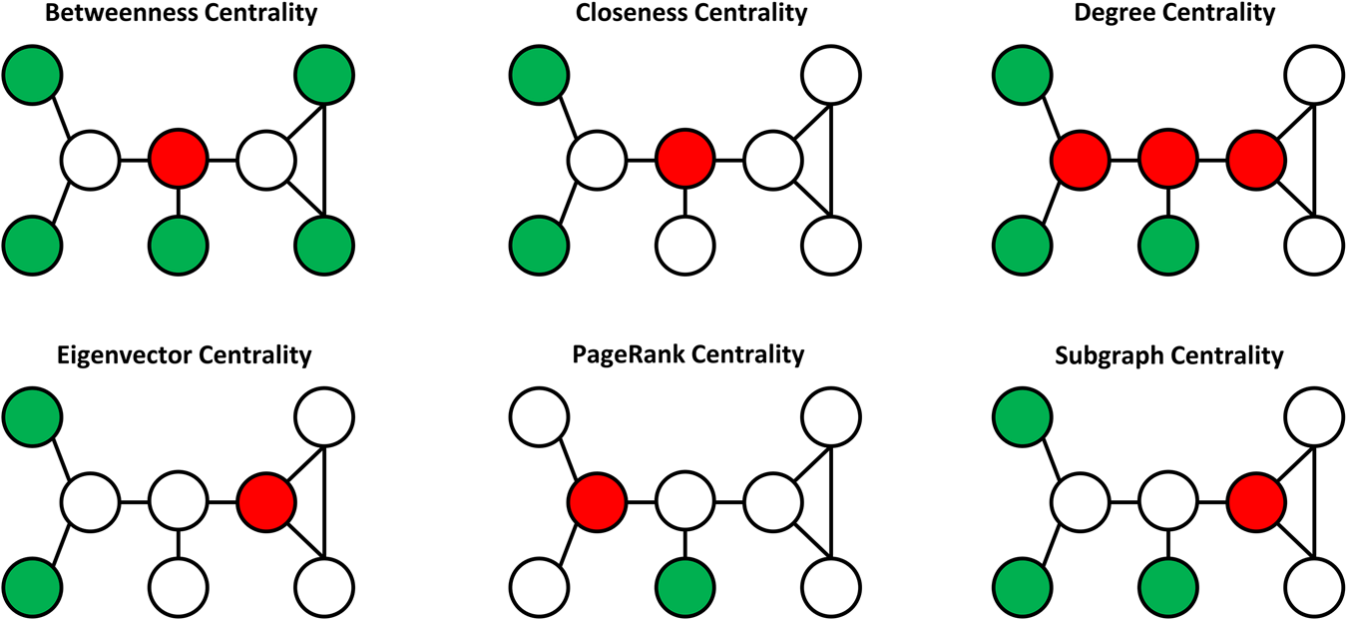
Small network illustrates the distinct characteristics of the centrality indices studied here. Nodes colored red or green to represent the highest and lowest values for each measure, respectively.

To illustrate the distinct characteristics of these measures, we constructed a small network of eight nodes and eight edges, visualizing the highest and lowest values for each centrality (Fig. 7).

### Description of stochastic edge removal methods

We developed six edge removal methods to simulate different types of network disruptions:

1. Random Edge Removal (RER): This method randomly selects an edge from the graph and removes it. Each edge has an equal probability of being selected for deletion, regardless of the degree of the nodes it connects or their position in the network. This approach introduces unbiased randomness, allowing edges to be removed without favoring specific nodes or structures. Let *G* = (*V*, *E*) be the graph, where *V* is the set of nodes and *E* is the set of edges. In this method, each edge in the graph is assigned an equal probability of being removed. Let *e* be an edge in *E*. The probability *P(e)* is given by:
  1 $$P\left(e\right)=\frac{1}{\left|E\right|},\,e\in E$$
2. Highly Connected Edge Removal (HCER): This method prioritizes nodes with a high degree for edge removal. It first selects a high-degree node and then randomly chooses one of its edges to delete. By focusing on hubs, this method simulates targeted disruptions to highly connected nodes. In this method, the probability of removing an edge *e_uv* connecting nodes *u* and *v* is proportional to the sum of their degrees. Assign edge removal probabilities:
  2 $$P\left({e}_{{uv}}\right)\propto p\left(u\right)+p\left(v\right),{where}\,p\left(v\right)={\rm{deg}} (v)$$
3. Lowly Connected Edge Removal (LCER): This approach favors nodes with a low degree for edge removal. It selects a low-degree node and randomly removes one of its connected edges. By targeting less connected nodes, this method reduces edge density gradually, with minimal impact on core network structures. The probability of removing an edge *e_uv* is proportional to the inverse of the node degrees adjusted by the maximum degree.
4. Combined Edge Removal (CER): This method targets nodes at the extremes of the degree distribution, either high or low. It first selects either a high-degree or low-degree node (avoiding nodes with mid-range degrees) and then removes one of its edges randomly. This dual-targeting approach can mimic scenarios where both central and peripheral nodes are impacted, balancing effects across the network. A function *h(x)* measures the distance from the mean degree to determine removal probability.
  3 $$h\left(x\right)=\frac{1}{1+\left|{\rm{x}}-{\rm{mean}}\left({\rm{deg}} \right)\right|},P\left({e}_{{uv}}\right)\propto h\left({\rm{deg}} \left(u\right)\right)+h({\rm{deg}} (v))$$
5. Randomized Node-based Edge Removal (RNBER): In this method, each node is assigned a random probability value, which influences its likelihood of having an edge removed. Nodes are selected according to these random values, and an edge is removed at random from each chosen node. This method introduces stochastic variation at the node level, resulting in an unpredictable pattern of edge deletions across the network. Each node is assigned a random value between 0 and 1. The probability of removing an edge *e_uv* is determined by the product of these random values for the nodes *u* and *v*.
  4 $$P\left({e}_{{uv}}\right)=r\left(u\right).r\left(v\right),\,r(v)\sim {Uniform}(0,1)$$
6. Random ealk Edge Removal (ReER): Removes edges based on their probability of being selected during a random walk, mimicking biased information collection due to chains of experimental attention (collecting information on a node triggers research on the direct neighbors of this node). The removal probability is proportional to the sum of the values of the adjacent nodes.
  5 $$P\left({e}_{{uv}}\right)={PR}\left({\rm{u}}\right)+{PR}\left({\rm{v}}\right),\,P\left({e}_{{uv}}\right)=\,\frac{P({e}_{{uv}})}{\sum P(e)}$$

## Supplementary information

Supplementary Information

## Data Availability

All data used in this study are publicly available from the GitHub repository: https://github.com/alisalehzadeh-yazdi/centrality-robustness-simulator. This repository contains the dataset necessary to interpret, replicate, and build upon the findings reported in this article.

## Code availability

Computer code is available from the GitHub repository https://github.com/alisalehzadeh-yazdi/centrality-robustness-simulator.
