# Supplementary material for: Assessing the impact of sampling bias on node centralities in synthetic and biological networks
Source: NPJ Syst Biol Appl. 2025 May 15;11:47. doi: 10.1038/s41540-025-00526-w (PMC12081662; doi:10.1038/s41540-025-00526-w)
Supplement: Supplementary file 1 — Supplementary Information [file 41540_2025_526_MOESM1_ESM.pdf]

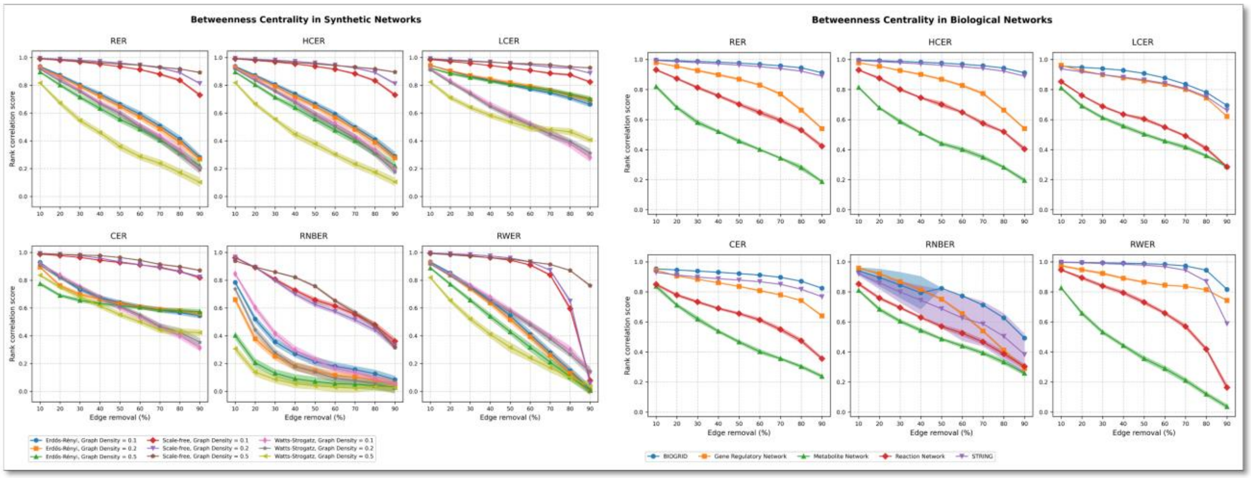
Betweeness Centrality in Synthetic Networks
RER
HCER
LCER
CER
RNBER
RWER
Rank correlation score
Edge removal (%)
Erdős-Rényi, Graph Density = 0.1
Erdős-Rényi, Graph Density = 0.2
Erdős-Rényi, Graph Density = 0.5
Scale-free, Graph Density = 0.1
Scale-free, Graph Density = 0.2
Scale-free, Graph Density = 0.5
Watts-Strogatz, Graph Density = 0.1
Watts-Strogatz, Graph Density = 0.2
Watts-Strogatz, Graph Density = 0.5
Betweeness Centrality in Biological Networks
BIOGRID
Gene Regulatory Network
Metabolite Network
Reaction Network
STRING

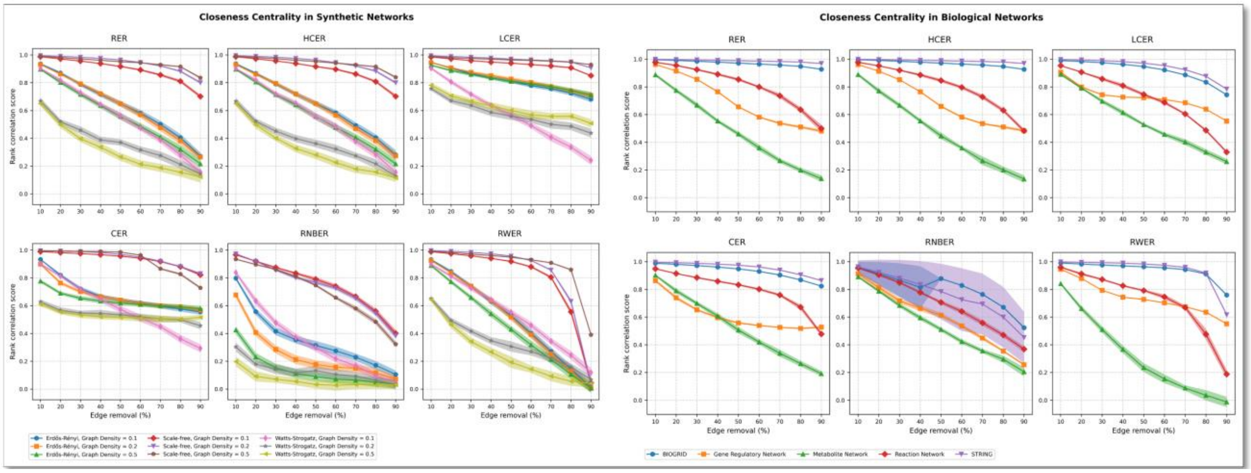
Closeness Centrality in Synthetic Networks
RER
HCER
LCER
CER
RNBER
RWER
Rank correlation score
Edge removal (%)
Erdős-Rényi, Graph Density = 0.1
Erdős-Rényi, Graph Density = 0.2
Erdős-Rényi, Graph Density = 0.5
Scale-free, Graph Density = 0.1
Scale-free, Graph Density = 0.2
Scale-free, Graph Density = 0.5
Watts-Strogatz, Graph Density = 0.1
Watts-Strogatz, Graph Density = 0.2
Watts-Strogatz, Graph Density = 0.5
Closeness Centrality in Biological Networks
BIOGRID
Gene Regulatory Network
Metabolite Network
Reaction Network
STRING

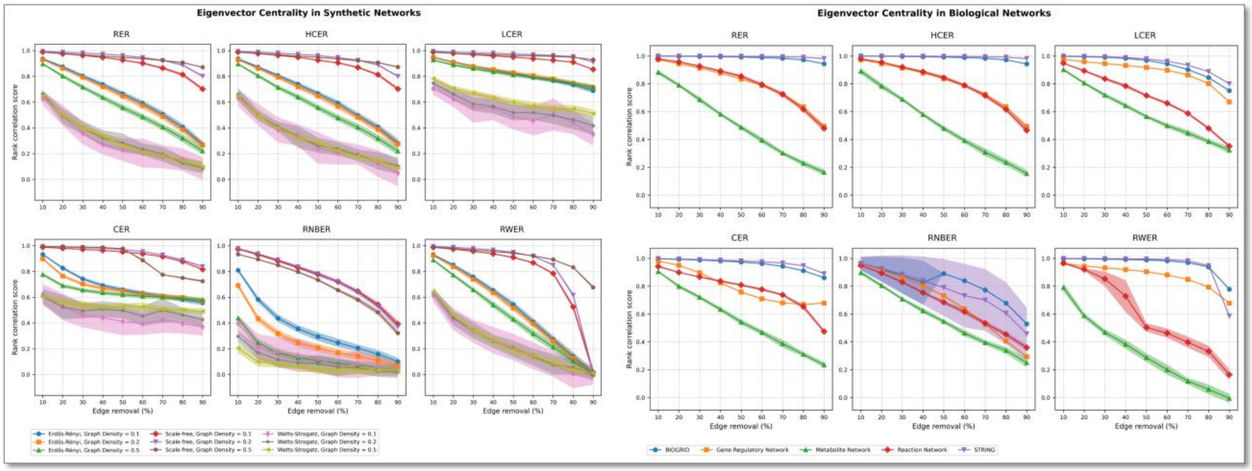

Eigenvector Centrality in Synthetic Networks
RER
HCER
LCER
CER
RNBER
RWER
Rank correlation score
Edge removal (%)
Erdős-Rényi, Graph Density = 0.1
Erdős-Rényi, Graph Density = 0.2
Erdős-Rényi, Graph Density = 0.5
Scale-free, Graph Density = 0.1
Scale-free, Graph Density = 0.2
Scale-free, Graph Density = 0.5
Watts-Strogatz, Graph Density = 0.1
Watts-Strogatz, Graph Density = 0.2
Watts-Strogatz, Graph Density = 0.5
Eigenvector Centrality in Biological Networks
BIOGRID
Gene Regulatory Network
Metabolite Network
Reaction Network
STRING

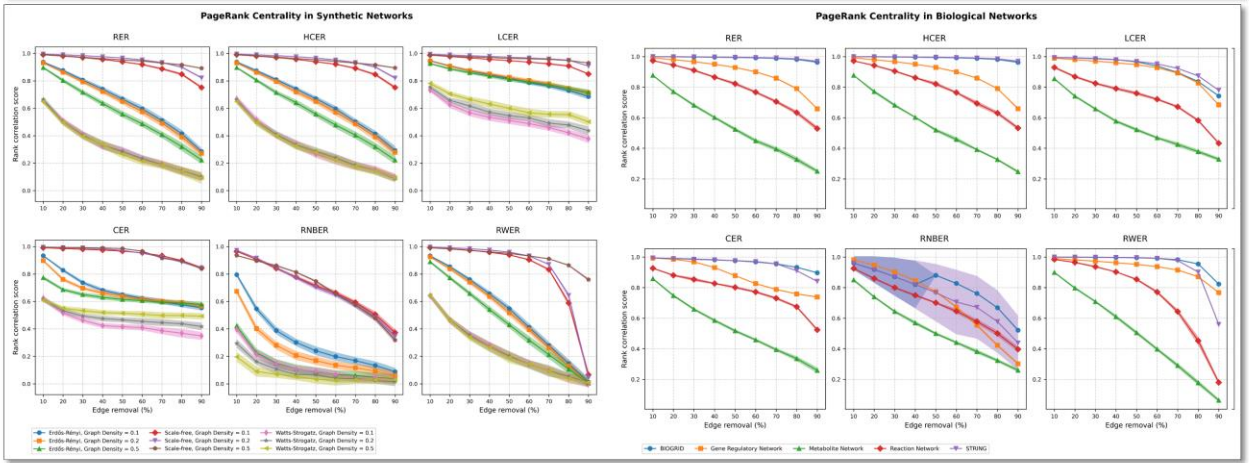

PageRank Centrality in Synthetic Networks
RER
HCER
LCER
CER
RNBER
RWER
Rank correlation score
Edge removal (%)
Erdős-Rényi, Graph Density = 0.1
Erdős-Rényi, Graph Density = 0.2
Erdős-Rényi, Graph Density = 0.5
Scale-free, Graph Density = 0.1
Scale-free, Graph Density = 0.2
Scale-free, Graph Density = 0.5
Watts-Strogatz, Graph Density = 0.1
Watts-Strogatz, Graph Density = 0.2
Watts-Strogatz, Graph Density = 0.5
PageRank Centrality in Biological Networks
BIOGRID
Gene Regulatory Network
Metabolite Network
Reaction Network
STRING

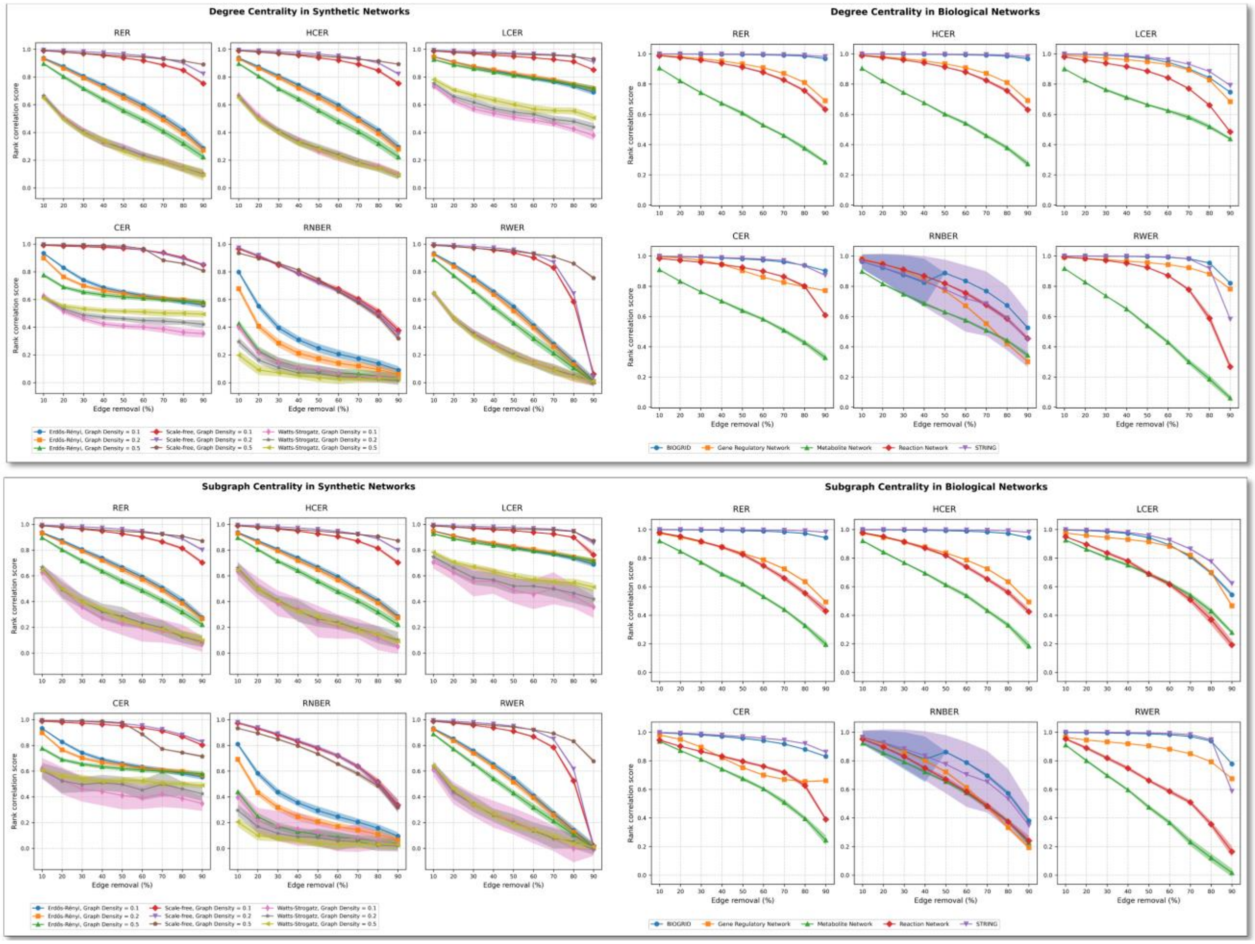

Supplementary Figure 1. Rank correlation score of all six centrality across different edge removal methods

Here, we present the results in six panels, each corresponding to a particular centrality measure. To facilitate interpretation, we further divide each panel into two parts, showcasing the results for synthetic and biological networks separately. Within each panel, we illustrate the changes in rank correlation scores under various edge removal scenarios, highlighting the impact of each removal scenario, network structure, network density, and biological network type on these changes.

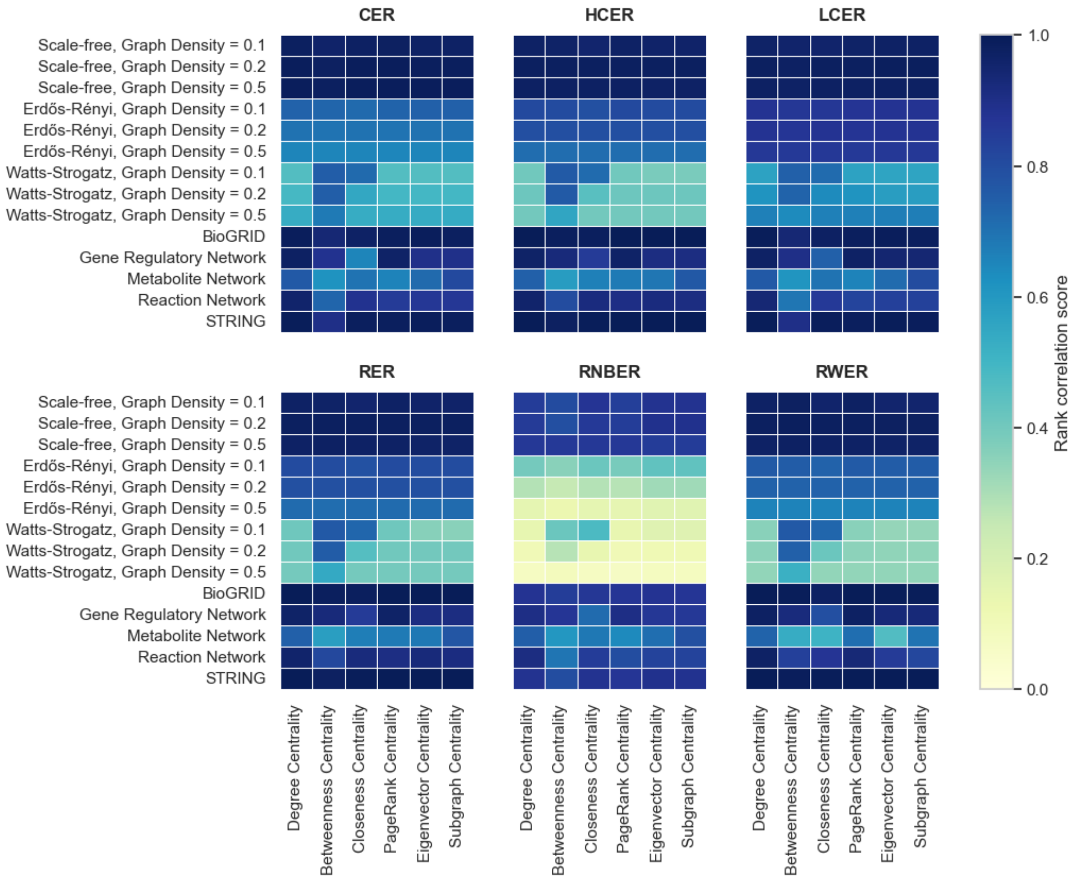

Supplementary Figure 2. Heatmap visualization of rank correlation scores for 30% edge removal across various network types and centrality measures

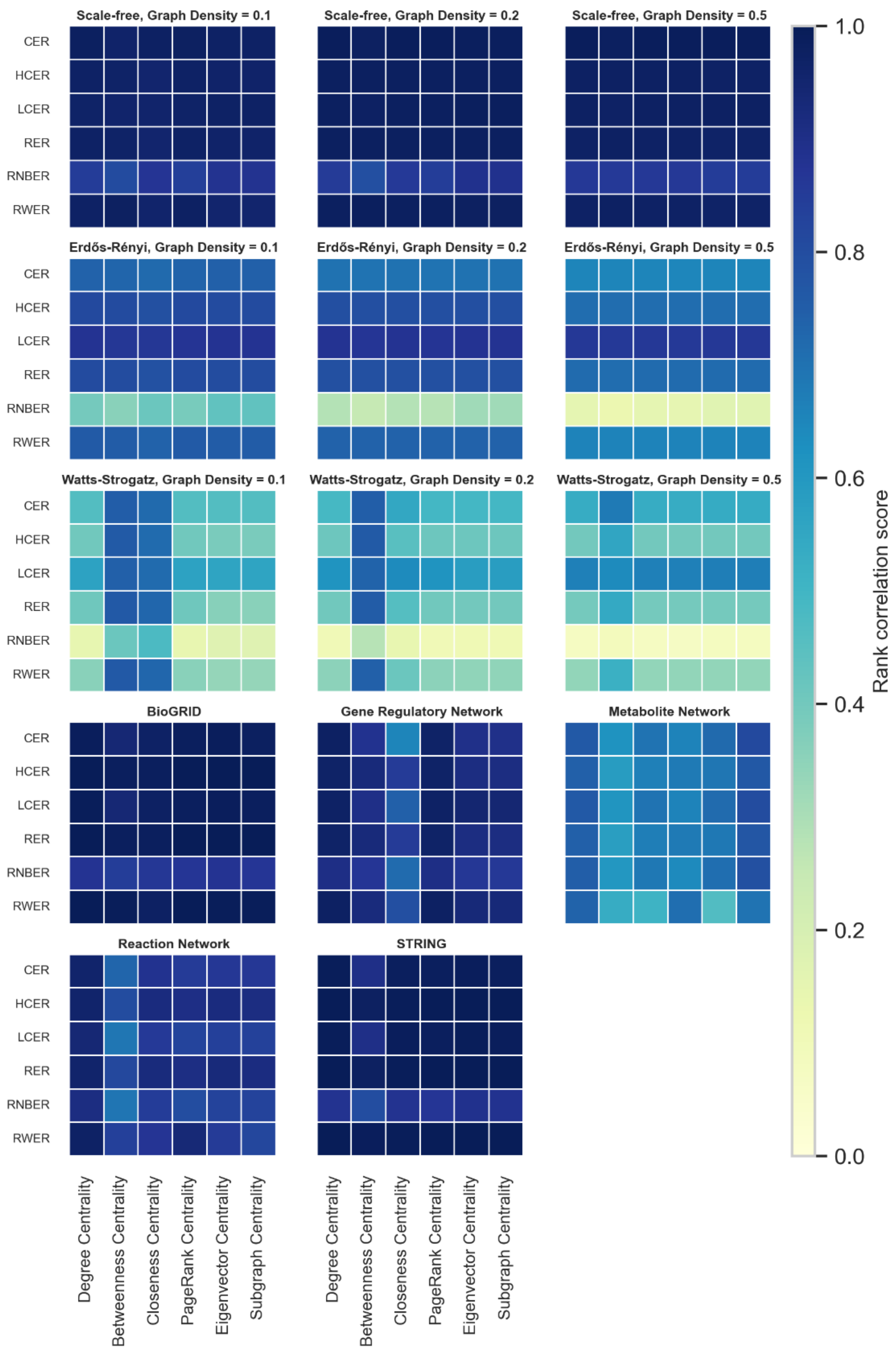

Supplementary Figure 3. Heatmap visualization of rank correlation scores for 30% edge removal across various network types and centrality measures

To simplify interpretation, we generated a heatmap visualization (Supplementary Figure 2 and Supplementary Figure 3) presenting rank correlation scores for 90% edge removal across all cases.

Supplementary Note 1

This file provides the detailed results of the stable nodes (colored green in Figure 7) analysis performed for each centrality measure under different stochastic edge removal methods. For each method, the stability threshold (defined as the 25th percentile of the standard deviation of ranks) is reported, along with the list of stable nodes. Each stable node is presented with its metabolite name, average rank, and standard deviation of ranks across all perturbation levels. The results are organized by centrality measure (degree, betweenness, closeness, PageRank, eigenvector, and subgraph centrality) and by edge removal method (RER, HCER, LCER, CER, RNBER, RWER).

STABLE NODES ANALYSIS for RER

==

DEGREE CENTRALITY: Stability threshold: 10.471

Metabolite	Average Rank	Std Deviation
sM_glu__L_c	2	1.25
sM_nh4_c	2.89	2.47
sM_coa_x	4.67	4.22
sM_accoa_x	5.67	2.4
sM_akg_c	9.78	9.05
sM_gdp_c	11.44	4.95
sM_gln__L_c	13.78	4.73
sM_12dgr_SC_c	19.56	8.92

BETWEENNESS CENTRALITY: Stability threshold: 18.070

Metabolite	Average Rank	Std Deviation
sM_akg_c	14.78	8.31

CLOSENESS CENTRALITY: Stability threshold: 167.730

Metabolite	Average Rank	Std Deviation
sM_glu__L_c	13.89	18.59
sM_nh4_c	42.22	77.28
sM_akg_c	43.33	27.74
rR_ASPTA	66.88	47.46
sM_gln__L_c	68.78	59.98
rR_GLUDxi	82.88	82.21
sM_hdcoa_c	162.44	128.42
sM_ac_c	195.11	130.3

PAGERANK CENTRALITY: Stability threshold: 200.443

Metabolite	Average Rank	Std Deviation
sM_nh4_c	2.11	1.85
sM_glu__L_c	2.11	0.87
sM_coa_x	6.22	5.49
sM_accoa_x	9.33	4.67
sM_akg_c	10	9.48
sM_gdp_c	10.67	3.89
sM_gln__L_c	13.11	5.7
sM_pyr_c	14.89	11.27
sM_12dgr_SC_c	20.22	8.35
sM_pi_m	24.11	29.85
sM_malcoa_c	28.33	32.09
sM_h2o2_c	30.22	25.11
sM_glu__L_m	32.33	28.19
sM_atp_x	37.44	37.41
sM_accoa_m	44.44	43.98
sM_h_n	46.78	48.18
sM_h_r	54.44	30.19
sM_amet_c	57.67	71.48
sM_hdcea_c	61	25.04
sM_o2_m	102.33	74.43
sM_tyr__L_c	104.89	91.07
sM_dhap_c	111.67	146.72
sM_for_c	112.78	149.72
sM_acald_c	119.67	139.5
rR_ERGSTESTH_SCe	130.56	51.27
sM_3mbald_c	132.11	56.78
sM_ACP_c	156.11	96.68
sM_ocdcea_c	174.22	131.21
rR_AGAT_SC	177.78	101.53
sM_hdcea_e	190	98.89
rR_THZPSN1_SC	209.78	154.41
sM_2phetoh_c	215	156.96
sM_2mbtoh_c	231.56	176.66
rR_FECOSTAT_SC	404.89	194.56

EIGENVECTOR CENTRALITY: Stability threshold: 123.001

Metabolite	Average Rank	Std Deviation
sM_nh4_c	72.11	106.23

SUBGRAPH CENTRALITY: Stability threshold: 130.895

Metabolite	Average Rank	Std Deviation
sM_glu__L_c	2	1.25
sM_nh4_c	3.89	3.07
sM_coa_x	4.22	4.78
sM_accoa_x	6.22	2.2
sM_akg_c	15	20.34
sM_atp_x	16.56	10.31
sM_gln__L_c	18	10.89
sM_gdp_c	23.44	19.55
sM_pyr_c	33.67	22.34
sM_12dgr_SC_c	35	17.38
sM_hdcea_c	36.22	18.16
sM_malcoa_c	40.44	33.7
sM_accoa_c	42.11	42.65
sM_amp_x	42.33	23.08
sM_pmtcoa_c	52.78	93.03
rR_AGAT_SC	53.33	46.61
sM_h_n	60.44	42.95
sM_ocdcea_c	64	38.04
sM_glu__L_m	68.33	32.69
sM_h2o2_c	71	48.94
sM_tdcoa_c	72.22	121.13
rR_EPISTAT_SC	75.44	98.24
sM_amet_c	80.22	71.42
sM_hdcea_e	93.11	47.33
rR_ERGSTESTH_SCe	95.33	36.67
rR_FAO181p_odd	101.11	49.65
sM_ocdycacoa_c	101.33	87.37
sM_h_r	108.33	95.58
rR_FACOAL240p	111.33	121.52
rR_THZPSN1_SC	111.44	100.11
sM_f6p_c	154.22	124.64
rR_GLNS	161.11	86.04
sM_tyr__L_c	171.78	116.8
rR_ACACT8p	207.89	110.13

STABLE NODES ANALYSIS for HCER

==

DEGREE CENTRALITY: Stability threshold: 11.829

Metabolite	Average Rank	Std Deviation
sM_nh4_c	2	0.67
sM_coa_x	4.11	1.2
sM_glu__L_c	4.78	9.64
sM_akg_c	11.22	8.15
sM_pyr_c	11.78	8.32
sM_accoa_c	12.78	8.66
sM_accoa_x	15.89	10.68
sM_amet_c	19.11	8.09

BETWEENNESS CENTRALITY: Stability threshold: 62.968

Metabolite	Average Rank	Std Deviation
sM_coa_x	56.89	43.84

CLOSENESS CENTRALITY: Stability threshold: 127.975

Metabolite	Average Rank	Std Deviation
sM_nh4_c	13.22	11.34
sM_asp__L_c	33.67	37.85
rR_GLUDy	55	69.4
rR_GLUN	69.62	69.86
rR_IPCS324_SC	138.78	87.84
rR_AGAT_SC	151.56	97.85
sM_dcacoa_c	248.38	114.08
sM_gdp_c	273.78	114.78

PAGERANK CENTRALITY: Stability threshold: 301.887

Metabolite	Average Rank	Std Deviation
sM_nh4_c	1.67	0.82
sM_glu__L_c	4.33	7.67
sM_coa_x	5.33	2.4
sM_akg_c	10.33	6.51
sM_accoa_c	13.56	10.3
sM_pyr_c	14.11	16.78
sM_amet_c	14.67	6.16
sM_gdp_c	15.33	10.07

sM_12dgr_SC_c	19.89	11.7
sM_accoa_x	24.89	20.11
sM_adp_m	33	33.78
sM_ptd1ino_SC_c	33.78	39.02
sM_atp_x	34.78	38.55
sM_f6p_c	38.22	18.44
sM_h2o2_c	41.11	63.96
sM_pi_m	45.22	91.32
sM_asp__L_c	58.44	80.52
sM_ttdca_c	58.56	28.37
sM_acald_c	59.33	81.61
sM_ctp_c	60.22	21.58
sM_gly_c	72.33	83.02
sM_for_c	72.56	94.89
rR_PLBP1I_SCe	97.33	68.36
sM_stcoa_c	109.11	60.95
sM_pmtcoa_c	115.11	90.33
sM_ibutoh_c	142	117.41
sM_cys__L_c	159.22	187.97
sM_oaa_c	177.67	200.76
sM_iamoh_c	180.67	197.72
sM_ocdcea_c	193	279.77
rR_TAGL_SC	253.89	215.22
sM_2mppal_c	267.44	261.34
rR_LPCAT_SC	280.89	248.86
sM_dadp_c	436.89	270.19

EIGENVECTOR CENTRALITY: Stability threshold: 186.683

Metabolite	Average Rank	Std Deviation
sM_atp_x	255.67	180.54

SUBGRAPH CENTRALITY: Stability threshold: 199.120

Metabolite	Average Rank	Std Deviation
sM_nh4_c	2.11	0.74
sM_coa_x	4.22	1.87
sM_glu__L_c	9	21.57
sM_atp_x	16.44	13.21
sM_akg_c	17.11	19.65
sM_pyr_c	20.78	12.37
sM_gdp_c	25.44	20.09

sM_ptd1ino_SC_c	25.78	16.19
sM_12dgr_SC_c	29.89	24.14
sM_accoa_x	31.89	26.53
sM_accoa_c	34.11	27.72
sM_ttdca_c	39.67	24.51
sM_amet_c	40.78	26.85
sM_asp__L_c	45	27.76
rR_GAT2_SC	62.56	89.4
sM_pi_m	75	95.44
rR_AGAT_SC	75.56	102.5
sM_h2o2_c	77.78	52.07
sM_ocdycacoa_c	78.89	44.49
rR_PLBP1I_SCe	89.89	70.08
sM_ocdcea_c	95.22	83.69
rR_THZPSN2_SC	103	135.34
rR_FACOAL100p	110	103.59
sM_ppi_x	112.78	197.94
sM_hdcea_e	115	48.38
sM_f6p_c	117.56	59.53
sM_ctp_c	131.56	58.75
sM_ddcacoa_c	156.22	196.58
sM_glycogen_c	164.78	107.14
rR_ERGSTESTH_SCe	165.89	167.37
sM_acald_c	172.44	184.07
sM_for_c	173	113.76
sM_hcys__L_c	186.78	131.56
rR_IPCS324_SC	226.22	149.93

STABLE NODES ANALYSIS for LCER

===

DEGREE CENTRALITY: Stability threshold: 3.991

Metabolite	Average Rank	Std Deviation
sM_glu__L_c	1.56	0.5
sM_nh4_c	3	1.15
sM_coa_x	3.56	0.5
sM_akg_c	6.44	2.75
sM_pmtcoa_c	13	3.65

BETWEENNESS CENTRALITY: Stability threshold: 38.041

Metabolite	Average Rank	Std Deviation
sM_glu__L_c	2.67	1.89
sM_atp_x	26.22	22.97

CLOSENESS CENTRALITY: Stability threshold: 9.740

Metabolite	Average Rank	Std Deviation
sM_glu__L_c	2.33	0.94
sM_asp__L_c	7.44	4.17
sM_gln__L_c	8.78	4.08
sM_met__L_c	15.78	2.1
sM_pe_SC_c	25.67	8.72

PAGERANK CENTRALITY: Stability threshold: 34.250

Metabolite	Average Rank	Std Deviation
sM_glu__L_c	1.78	0.42
sM_nh4_c	2.33	1.05
sM_coa_x	3.89	0.31
sM_akg_c	6.11	1.85
sM_gdp_c	10.22	5.77
sM_pyr_c	10.56	4.62
sM_12dgr_SC_c	13.89	5.7
sM_amet_c	15	4.4
sM_accoa_x	15.67	8.56
sM_atp_x	20.33	9.09
sM_accoa_c	20.56	33.05
sM_malcoa_c	20.78	8.38
sM_pi_m	21.33	19.91

sM_h2o2_c	24.44	13.17
sM_glu__L_m	25.78	32.66
sM_ptd1ino_SC_c	27.56	22.18
sM_ac_c	30.89	21.53
sM_ACP_m	32.33	12.23
sM_pmtcoa_c	37.33	8.93
sM_tdcoa_c	40	12.06
sM_gly_c	44.33	31.22
sM_ser__L_c	44.67	32.87
sM_ahcys_c	45.78	31.1
sM_utp_c	46	11.13
sM_trdrd_c	48.89	17.37
sM_cys__L_c	51.67	26.76
sM_ttdca_c	52	22.18
sM_succ_c	55.33	16.21
sM_odecoa_c	59	24.23
sM_atp_n	72.78	29.14
sM_hdcea_c	74.89	26.98
rR_PLBPC_SC	87.33	32.02
rR_GAT2_SC	110.22	32.35
rR_TRIGS_SC	134	28.43

EIGENVECTOR CENTRALITY: Stability threshold: 2.362

Metabolite	Average Rank	Std Deviation
sM_tdcoa_c	3.56	1.64
sM_pmtcoa_c	3.89	2.23
rR_TRIGS_SC	11.22	1.69

SUBGRAPH CENTRALITY: Stability threshold: 25.830

Metabolite	Average Rank	Std Deviation
sM_glu__L_c	1.44	0.5
sM_coa_x	3.33	1.89
sM_tdcoa_c	5.78	1.23
sM_nh4_c	6	3.23
sM_pmtcoa_c	6.56	2.83
sM_hdcoa_c	9.56	4.03
sM_stcoa_c	10	3.4
sM_odecoa_c	10.11	5.72
rR_GAT1_SC	11	3.89
rR_GAT2_SC	13.22	2.1

rR_LPCAT_SC	13.33	2.62
rR_TRIGS_SC	15.11	2.13
sM_akg_c	15.33	7.18
sM_atp_x	16.44	7.26
rR_AGAT_SC	17.56	17.05
sM_accoa_x	21.67	13.79
rR_ZYMSTAT_SC	23.11	5.17
rR_LANOSTAT_SC	23.56	6.93
sM_gln__L_c	25	15.08
rR_FECOSTAT_SC	26.33	19.09
rR_EPISTAT_SC	26.67	21.43
sM_ocdycacoa_c	26.89	6.82
sM_ttdca_c	32.33	10.12
sM_12dgr_SC_c	35.44	8.47
sM_hdca_c	36.22	17.07
sM_asp__L_c	37.22	17.17
sM_gdp_c	39.67	18.51
sM_pyr_c	41.11	14.61
sM_ptd1ino_SC_c	46.56	18.73
sM_ocdca_e	61.67	21.98
rR_FECOSTESTH_SC	64.78	22.3
sM_amet_c	75.67	20.2
sM_met__L_c	102.33	23.62
sM_h2o2_c	120	17.32

STABLE NODES ANALYSIS for CER

==

DEGREE CENTRALITY: Stability threshold: 6.652

Metabolite	Average Rank	Std Deviation
sM_glu__L_c	1.56	0.83
sM_nh4_c	2.22	1.03
sM_coa_x	3.78	0.42
sM_accoa_x	7.11	1.45
sM_pyr_c	9.44	1.83
sM_malcoa_c	11.22	3.99
sM_tdcoa_c	14.78	6.01

BETWEENNESS CENTRALITY: Stability threshold: 12.372

Metabolite	Average Rank	Std Deviation
sM_glu__L_c	2.44	1.07
sM_coa_x	20.22	11.84

CLOSENESS CENTRALITY: Stability threshold: 27.689

Metabolite	Average Rank	Std Deviation
sM_glu__L_c	2.11	0.74
sM_gln__L_c	17	19.11
sM_leu__L_c	30.89	11.28
rR_VALTA	50.56	21.86
rR_EHGLAT	52.33	17.33
rR_GLU5K	67.38	26.8
rR_GLUTRS	67.67	26.86

PAGERANK CENTRALITY: Stability threshold: 149.741

Metabolite	Average Rank	Std Deviation
sM_nh4_c	1.67	0.67
sM_glu__L_c	1.89	0.87
sM_coa_x	4.22	0.42
sM_pyr_c	9	2.21
sM_accoa_x	12	3.86
sM_accoa_c	12.44	18.26
sM_malcoa_c	15.22	6.73
sM_12dgr_SC_c	18.78	7.28
sM_ptd1ino_SC_c	21.44	12.45

sM_h_n	23	7.94
sM_atp_x	24.11	11.01
sM_gtp_c	27.78	11.49
sM_gln__L_c	29.22	41.61
sM_ahcys_c	31.22	15.75
sM_tdcoa_c	34	14.04
sM_asp__L_c	34.11	40.55
sM_h2o2_c	34.33	27
sM_gly_c	34.44	18.82
sM_glu__L_m	42.11	56.97
sM_utp_c	60.44	62.57
sM_acald_c	71.67	96.96
rR_THZPSN1_SC	79.78	38.01
sM_ocdca_c	83.33	52.89
sM_malACP_m	88.33	71.74
rR_PLBP1I_SCe	91.33	51.78
sM_adp_n	93.67	40.03
sM_r5p_c	106.22	102.91
sM_gdpmann_c	114.22	65.35
sM_o2_m	124.67	143.61
sM_fum_c	129.44	100.56
sM_q6h2_m	139.89	101.55
rR_EPISTESTH_SC	154.67	84.29
rR_LPCAT_SC	177.56	107.4
rR_EPISTESTH_SCe	189	137.13

EIGENVECTOR CENTRALITY: Stability threshold: 26.888

Metabolite	Average Rank	Std Deviation
sM_glu__L_c	1.11	0.31
rR_GLU5K	42.62	18.53

SUBGRAPH CENTRALITY: Stability threshold: 66.279

Metabolite	Average Rank	Std Deviation
sM_glu__L_c	1.33	0.67
sM_coa_x	3.11	0.87
sM_nh4_c	3.56	0.68
sM_accoa_x	8.89	4.93
sM_tdcoa_c	9.44	3.56
sM_gln__L_c	16.67	6.07
sM_atp_x	19.78	21.18

sM_pyr_c	24.11	12.49
sM_akg_c	24.11	35.41
rR_AGAT_SC	30.22	26.04
sM_stcoa_c	33.89	52.72
sM_hdcoa_c	35.56	52.59
sM_asp__L_c	37.11	25.6
sM_malcoa_c	37.89	24.92
sM_accoa_c	39.22	36.81
sM_12dgr_SC_c	39.89	17.76
sM_hdca_c	49.44	35.72
rR_LPCAT_SC	51.22	59.85
sM_gly_c	54.56	37.33
rR_LANOSTAT_SC	55.11	43.52
rR_THZPSN1_SC	61.67	33.47
sM_ocdca_c	70.11	48.71
rR_FACOAL141p	71.78	33.87
sM_cys__L_c	74.44	39.73
sM_h_r	81	36.39
rR_EPISTESTH_SC	82.22	31.48
rR_TAGL_SC	87.11	48.14
rR_EHGLAT	92.56	31.12
sM_h2o2_c	105.44	49.52
sM_leu__L_c	105.89	46.3
sM_ocdycacoa_c	110.56	63.54
sM_ump_c	125.44	63.34
sM_adp_n	161.89	47.94
rR_DCMPDA	184.11	61.88

STABLE NODES ANALYSIS for RNBER

==

DEGREE CENTRALITY: Stability threshold: 4.955

Metabolite	Average Rank	Std Deviation
sM_nh4_c	2.44	1.34
sM_glu__L_c	2.56	1.89
sM_coa_x	4.22	2.94
sM_akg_c	7.67	4.88
sM_accoa_x	8.67	3.71
sM_gdp_c	11.44	4.14

BETWEENNESS CENTRALITY: Stability threshold: 26.476

Metabolite	Average Rank	Std Deviation
sM_glu__L_c	2.89	2.51
sM_atp_x	24.33	13.54

CLOSENESS CENTRALITY: Stability threshold: 27.579

Metabolite	Average Rank	Std Deviation
sM_glu__L_c	9.11	14.28
sM_tyr__L_c	24.11	10.08
sM_tre_c	30.71	10.31
sM_dtmp_c	35.5	12.04
rR_TYRTAi	51.62	19.96
rR_GLUCYS	56.71	25.87

PAGERANK CENTRALITY: Stability threshold: 159.274

Metabolite	Average Rank	Std Deviation
sM_nh4_c	1.89	0.74
sM_glu__L_c	2.33	1.25
sM_coa_x	5.22	3.97
sM_akg_c	6.33	2.11
sM_accoa_c	9.33	5.4
sM_gdp_c	10.11	3.28
sM_accoa_x	14.56	5.56
sM_glu__L_m	15.67	8.81
sM_malcoa_c	18	6.98
sM_pyr_c	23.56	24.6
sM_ac_c	27.89	23.12

sM_ahcys_c	28.22	23.77
sM_atp_x	30.78	14.31
sM_h2o2_c	32.22	24.3
sM_ttdca_c	39.33	19.74
sM_hdca_c	49.67	23.02
sM_gly_c	55.44	79.3
sM_stcoa_c	62.67	33.25
sM_malACP_m	69.44	32
sM_udp_c	71.44	87.28
sM_cer1_24_c	85.44	30.09
sM_adp_n	90.78	83.7
sM_ACP_c	92.44	48.99
sM_f6p_c	102.33	151.38
sM_ttdca_e	111	59.08
sM_ump_c	111.33	143.44
sM_hdcea_c	114.89	118.09
rR_GAT2_SC	128.11	71.55
sM_atp_n	137.67	127.7
rR_AGAT_SC	165.11	120.06
sM_ocdycacoa_c	183	110.02
rR_RNDR3n	254.78	123.96
sM_ddcacoa_c	267.89	140.76
sM_3mob_c	278.44	148.2

EIGENVECTOR CENTRALITY: Stability threshold: 31.347

Metabolite	Average Rank	Std Deviation
sM_pmtcoa_c	25.11	31.28
rR_LPCAT_SC	30.22	30.48

SUBGRAPH CENTRALITY: Stability threshold: 240.892

Metabolite	Average Rank	Std Deviation
sM_coa_x	11	19.12
sM_accoa_x	18.44	16.87
sM_pmtcoa_c	20.22	30.12
sM_stcoa_c	22.67	30.83
sM_glu__L_c	27.78	70.16
sM_hdcoa_c	30.33	33.6
rR_AGAT_SC	32.11	36.36
sM_akg_c	34.78	61
sM_ocdycacoa_c	36.11	26.49

sM_nh4_c	37.56	95.27
sM_odecoa_c	37.78	41.95
sM_ddcacoa_c	38.44	28
sM_dcacoa_c	46.67	26.6
sM_atp_x	52.89	84.02
sM_malcoa_c	61	27.46
sM_gln__L_c	63.11	97.68
sM_accoa_c	68.56	30.84
sM_pyr_c	70	38.63
sM_gly_c	72.22	35.89
sM_ahcys_c	74.33	51.4
sM_gdp_c	77.89	94.53
rR_ACSp	84	79.39
sM_ac_c	99.89	65.28
sM_amet_c	110.11	58.03
sM_gtp_c	129.78	88.12
sM_o2_m	152	79.75
sM_h2o2_c	174.89	121.31
sM_udp_c	175.44	153.44
sM_cys__L_c	179.67	233.07
sM_adp_x	183.22	105.72
sM_ACP_c	254.56	103.83
sM_cer1_24_c	296	139.18
sM_cer2_24_c	301.67	162.69
sM_utp_c	351.56	222.16

STABLE NODES ANALYSIS for RWER

==

DEGREE CENTRALITY: Stability threshold: 327.933

Metabolite	Average Rank	Std Deviation
sM_cer1_26_c	104.89	146.31
sM_q6h2_m	182.5	275.79
sM_focytc_m	184.71	107.75
rR_CYSTLp	204	128.34
sM_psphings_c	333.44	226.15
rR_3DH5HPBMTm	399.5	286
sM_pap_c	404.25	306.74
sM_thymd_c	410.33	248.49
sM_cit_x	445.67	301.56

BETWEENNESS CENTRALITY: Stability threshold: 506.513

Metabolite	Average Rank	Std Deviation
sM_octa_c	649.83	417.83

CLOSENESS CENTRALITY: Stability threshold: 461.735

Metabolite	Average Rank	Std Deviation
sM_cer1_26_c	374.78	386.15
sM_focytc_m	386.29	388.21
sM_cit_x	433.11	444.14
rR_FAS100	436.88	408.69
sM_q6h2_m	467.38	316.07
sM_mipc326_SC_c	517.5	387.75
sM_dadp_c	648.25	452.65
rR_CYSTLp	689.75	366.29
sM_4h2oglt_m	796.29	455.33

PAGERANK CENTRALITY: Stability threshold: 557.167

Metabolite	Average Rank	Std Deviation
sM_hcys__L_c	67	51.27
sM_cer1_26_c	102.11	113.52
sM_2obut_c	182.78	149.55
sM_accoa_m	231.44	410.81
sM_thymd_c	253.33	150.8
sM_glyc_c	278	349.93

sM_2mbald_c	300.11	198.73
rR_CYSTLp	324.44	506.15
sM_psphings_c	357.89	241.55
sM_ibutoh_c	378.33	520.33
sM_10fthf_c	396.89	491.07
rR_ALATRS	428.22	402.71
sM_iamac_c	432.89	319.36
sM_4abz_c	462	414.69
sM_ddcacoa_x	475.89	446.49
rR_23CAPPD	522.78	422.49
sM_ibutac_c	581.44	543.28
rR_LEUTRS	587.22	453.98
sM_akg_e	638.67	443.73
rR_GGTT	660	354.9
sM_pac_c	682.78	478.95
sM_2phetoh_m	715.44	392.49
sM_13BDglcn_e	726.33	522.21
rR_ADK4	811.89	446.82
sM_achms_c	824	492.17
sM_xtsn_e	852.78	532.2
rR_2MBTOHt	854.56	456.53
rR_MFAPS_SC	864.78	353.55
sM_idp_c	868	551.81
sM_tdcoa_x	912.89	521.08
rR_HEXDPtm	1050.33	543.69
rR_ALCD2irm	1082.78	541.37
sM_dd2coa_x	1140.89	526.74
sM_23camp_c	1307	547.78

EIGENVECTOR CENTRALITY: Stability threshold: 430.413

Metabolite	Average Rank	Std Deviation
----	----	----

SUBGRAPH CENTRALITY: Stability threshold: 621.897

Metabolite	Average Rank	Std Deviation
sM_cer1_26_c	171.78	227.25
sM_dca_c	197.67	137.21
sM_glx_c	334.33	182.42
sM_coa_r	352.22	467.53
sM_thymd_c	428.67	287.88

rR_CYSTLp	484.44	447.67
sM_psphings_c	540.11	355.71
sM_L2aadp_c	589.67	374.85
rR_AKGDbm	600.56	387.05
sM_cit_x	611.22	387.4
sM_fru_c	623.56	293.21
sM_dadp_c	643.33	620.94
rR_C24STRer	674.11	567.1
rR_CITtcp	687.11	462.64
rR_L_LACD2cm	687.89	609.99
rR_DOLPMMer	697.33	619.27
rR_TMDPP	701.78	436.21
rR_3DH5HPBMTm	719.78	512.93
sM_mipc326_SC_c	725	541.62
rR_CERS226	735.11	558.22
sM_myrsACP_m	756.33	585.1
sM_g6p_B_c	761.89	536.63
sM_nadph_r	837	573.13
sM_cyst__L_x	960.44	475.98
sM_hcys__L_x	1021.11	480.66
sM_cer1_24_r	1036	615.34
rR_SBTD_D2	1147.67	600.25
rR_GGTT	1232.22	548.33
rR_IAMACt	1258.11	608.37
sM_4abutn_c	1276.78	578.74
sM_cdpchol_c	1354.56	554.21
sM_s17bp_c	1369.22	561.09
sM_pendp_c	1408.22	567.08
sM_man_e	1468.33	585.16
